# The effect of using data palm waste fibers in plain concrete mixture by comparing three pre-treatment techniques

**DOI:** 10.1038/s41598-023-37860-0

**Published:** 2023-07-25

**Authors:** Dina R. M. Moawad, Rania Rushdy Moussa

**Affiliations:** 1 Environmental Sustainable Architecture Department, Faculty of Energy, The British University in Egypt (BUE), Cairo, Egypt; 2 Architecture Department, Faculty of Engineering, The British University in Egypt (BUE), Cairo, Egypt

**Keywords:** Environmental impact, Mechanical properties

## Abstract

The high rates of industrial and agricultural wastes and byproduct production produced several environmental concerns in addition to increasing the risks of contaminating natural resources. The amount of organic waste has been rising over the past few decades, but it has been poorly managed, especially in smaller countries where it is either burned, improperly disposed, or thrown in landfills. Moreover, the production of concrete generates a large amount of waste that has a negative impact on our planet even though it is an extremely significant building material. The potential of developing another plain concrete mixture sample with date palm mesh addition to create more environmentally friendly concrete with better qualities. In addition to using experimental quantitative research to measure the effectiveness of various concrete mixes after adding palm tree residues that underwent biological, chemical, & mechanical treatments, a theoretical qualitative method was also employed in this study to evaluate and identify the ideal mixture design. The prototypes' strength, density, thermal conductivity, slump, and absorption rate were all measured during the experiments. Having a lower heat conductivity and greater strength, the mechanically treated fibers with a 0.6% addition provided the best prototype, according to the research findings.

## Introduction

The increasing human population, consumption rate and human actions in countries all over the world resulted in the production an average of 38 billion metric tons of organic wastes per year^1^. Several researchers have studied various methods of using agricultural waste to develop environmentally friendly concrete mixtures with adequate strength and better mechanical qualities while having minimal environmental impact. Researchers have experimented methods of partially replacing cement content with palm wastes in order to manufacture environmentally friendly concrete to reduce the waste generated by cement production. The goal of this study is to reduce the environmental impact of palm wastes by using them as a component to the mixture of plain concrete, while also figuring out how to properly dispose and recycle organic wastes and byproducts and enhancing the qualities of plain concrete. In order to create the best sample, which has greater strengths and properties compared to the standard plain concrete but with a reduced thermal conductivity, a detailed assessment of organic wastes, cement, and plain concrete will be presented in this study, along with recommendations for combining them to reduce their environmental impact. The findings and results will help create a prototype sample that will be examined and contrasted with the control concrete mixture^2^.

### Organic wastes

**Figure 1:**
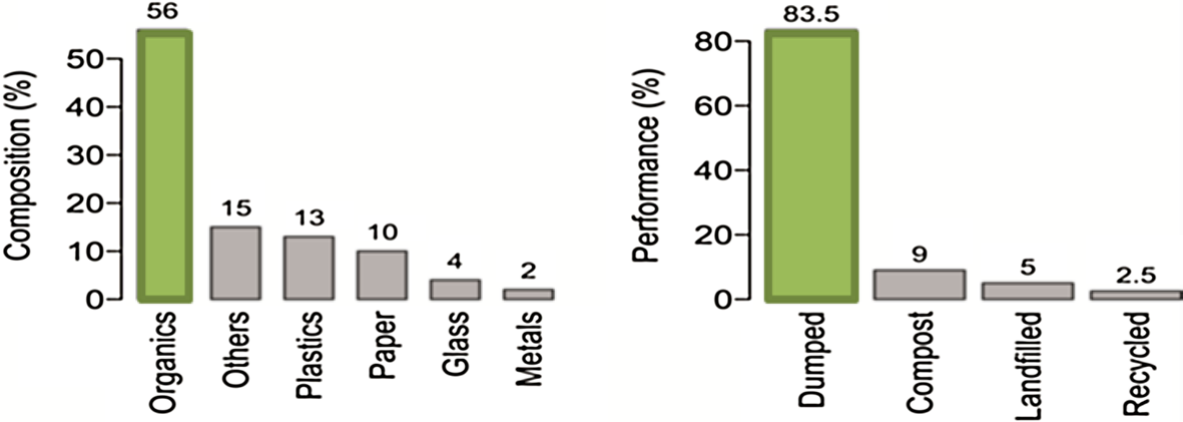
Composition (left) and performance (right) of MSW in Egypt as reported in 2010^5^.

Despite the existing 66 composting plants less than 25% of the organic waste in Egypt are being recycled^3^. The projection study of the solid waste generation in Fig. 1 below shows that by 2025 the organic wastes will exceed 30 million tons per year^4^. The improper discarding and burning of organic waste resulted in various environmental & health problems^5^.

There are three types of organic wastes which are the household organic waste, the agriculture organic waste, and the Industrial organic waste, studies show that 50% of organic wastes consists of agricultural wastes^6^.

**Figure 2:**
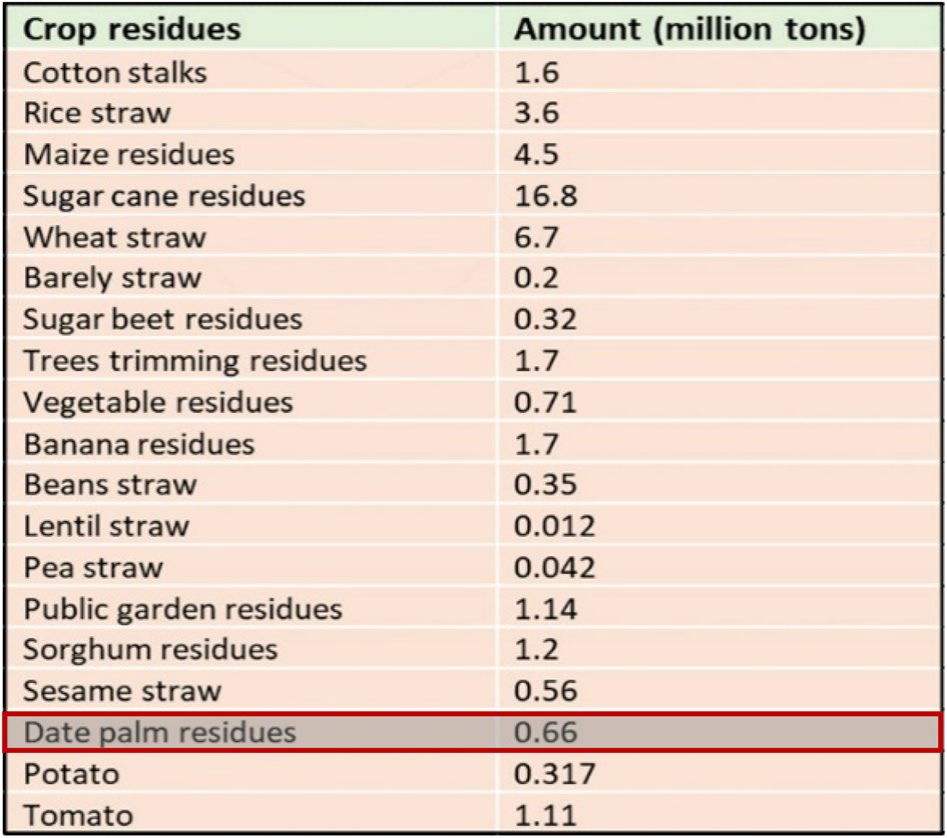
Types of agricultural waste and percentage of it in Egypt^8^.

The most important agriculture crops that are produced in Egypt are listed in Fig. 2 below along with their annual generation amounts. Egypt produces around 0.66 million tons of date palm residues^7^.

Egypt is the world's biggest producer of date palm fruit. Egypt's yearly date production increased by 70% in 2019, accounting for nearly 30,000 tons^9^. A single adult date palm tree when trimmed regularly can produce approximately 25 kg of waste per year^9^. In 2012 Egypt produced 1.47 million tons of dates which makes it the major date producer in the world since it presented one-fifth of the world’s production. The date palm rachis has a long history of being used in the handmade arts, ornaments, baskets, crates, and furniture^10^. The industrialized wooden strips that are made from the date palm rachis are used to make floorings that is an alternative to the timber floorings, and in the countryside, housings use the palm rachis as a covering material for their roofs.

**Figure 3:**
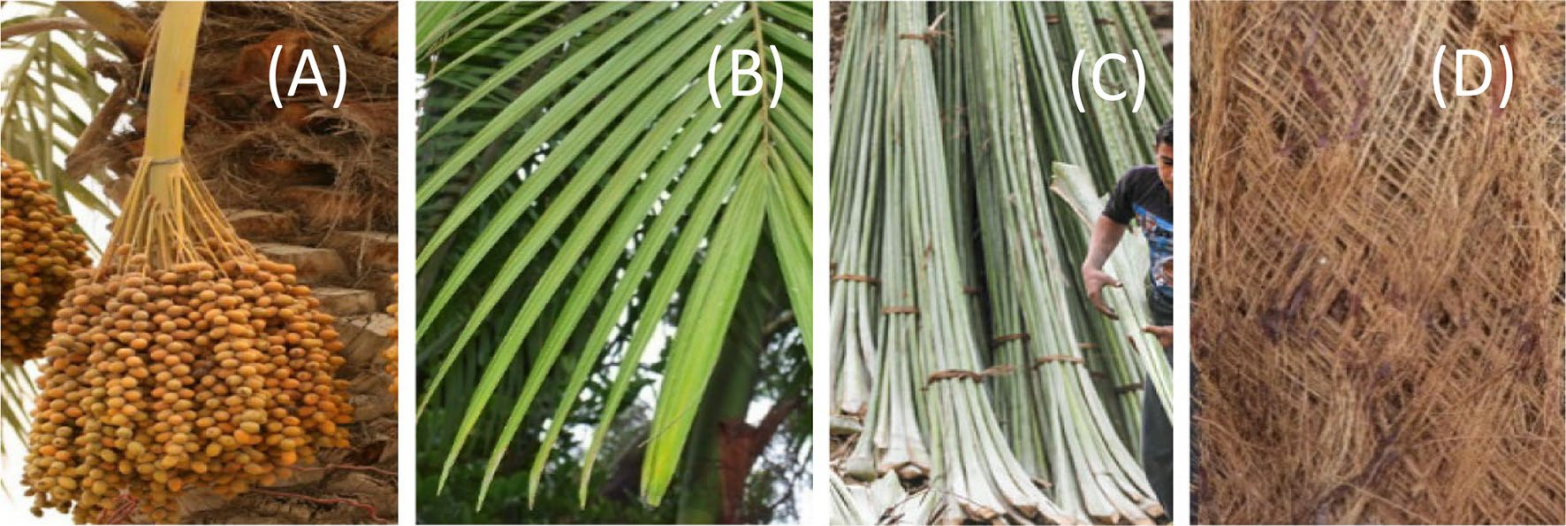
Date palm parts (**a**) spadix stems (**b**) leaflets (**c**) midribs (**d**) mesh.

Figure 3 illustrates the four parts of the date palm where fibers can be extracted: the mesh, the spadix stems, the midribs, and the leaflets. A date palm leaf is a compound leaf that includes three sections: the midrib, the midrib base, and the leaflets^11^.

**Figure 4:**
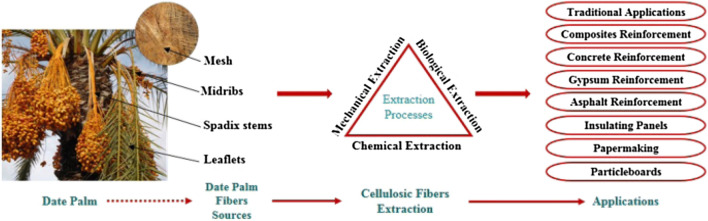
Utilizing palm residues in different industries^7^.

Comparing with the other date palm fiber species, male date palm surface fiber (MDPSF) has the best tensile strength which range between 170 and 300 MPa^12^. MDPSF can be obtained from the thin tissue that covers the top of the male plant and works as an insulator to protect the palm from the vagaries of weather^14^. Date palm fibers are usually treated to reduce the limitations and to improve their performance as shown in Fig. 4.

### Plain concrete

Concrete is a composite material composed of Portland cement, water, aggregates, and, in some cases, admixtures. The cement and water combine to form a paste that solidifies and bonds the aggregates^13^. The amount and properties of the materials used, as well as the method placing, finishing, and curing the concrete, all have a direct impact on its quality. Concrete is a versatile building material that can be used for a wide range of agricultural and residential purposes. It can withstand many acids, silage, milk, manure, fertilizers, water, fire, and abrasion with the right materials and techniques. Due to the usage of concrete as a structural material, strength is considered a very important property to have. Concrete's compressive strengths typically range from 2000 to 5000 pounds per square inch (psi), however for sometimes, concrete can be produced to withstand over 10,000 psi^14^.

Concrete is well known for its abilities to withstand compression; however, it is very week in tension which makes it very likely to shrink or crack since it becomes more fragile the more its strength increases. Accordingly, to increases its ability to withstand tensions stress steel reinforcement was used However, it is not preferred since it is very expensive, and its tremendous permeability can cause corrosions in it. Recently researchers proposed that polyvinyl alcohol (PVA) and polypropylene fibers can be used instead of steel fibers. However, the production of these fibers requires the use of unsustainable chemical compounds^14^.

There are variety of factors that can affect and control the properties of concrete such as the mix proportions that has a huge effect on the concrete's strength. Such as: Workability of Concrete,

Segregation of concrete, Bleeding in Concrete, Compressive Strength/Concrete grades, Tensile strength of concrete, and Shrinkage of concrete:

The main component of any concrete mixture is the concrete which is known to have major environmental impacts such as: Energy consumption which is the most serious environmental issue associated with cement and concrete production. Cement production is one of the most energy-intensive industries in the world. The manufacture of cement and the subsequent use of fuel for mining and transportations consumes around 1.758 kWh per ton of cement^15^. The heavy dependence on coal in industry contributes to extremely high emissions of CO_2_ and Sulphur, among other pollutants^15^.

CO_2_ emissions that are produced during the cement manufacturing process are generated from two different sources. The primary source is fossil fuels, which is around 3/4 tons CO_2_ per ton of cement and calcined into the cement kiln by lime process. The combustion of rotary kilns is the main cause. Around 5% of overall CO_2_ emissions are accountable to the cement industry. Apart from CO_2_, both cement and concrete production emit significant amounts of air pollution, with dust being the most visible pollutant which accounts for 360 pounds per ton of cement produced.^16^. Another environmental problem associated with cement and concrete production is water pollution. Water used for cleaning equipment is sometimes disposed through placing ponds at the batch plant, where the solids settle out^16^.

## Research methodology

**Figure 5:**
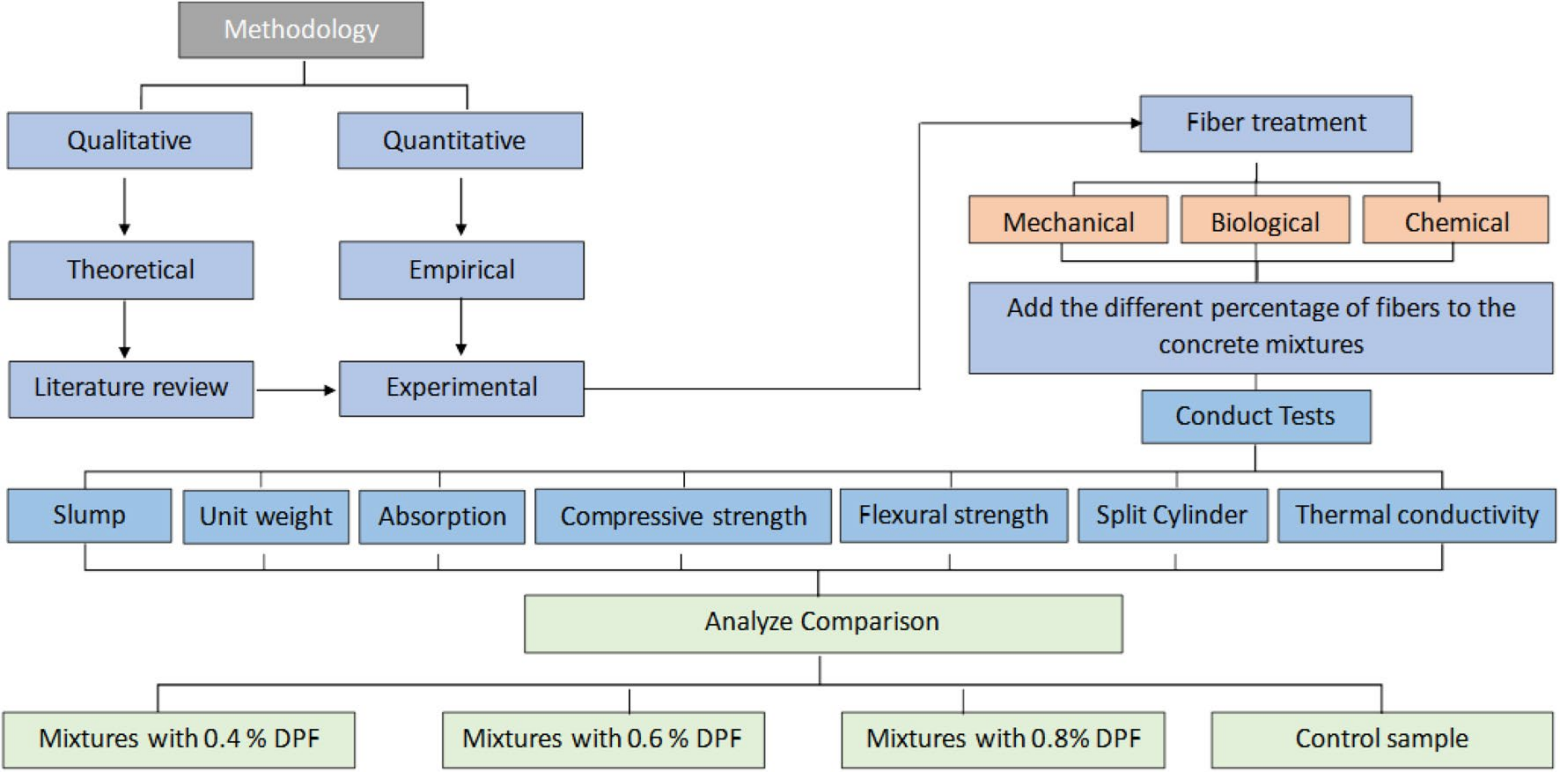
Research methodology (developed by author, 2023).

The research methodology is classified into two main categories based on the nature of this study: data gathering and data analysis approaches, as indicated in Fig. 5 below, this study is conducted using two methods: qualitative and quantitative.

### Materials and methods

The experiment in this study was divided into 2 phases, phase one is the fibers treatments, and phase two is the concrete mixing. The IUCN Policy Statement on Research Involving Species at Risk of Extinction and the Convention on the Trade in Endangered Species of Wild Fauna and Flora were both complied with, in the collection and use of plant samples for this study. In specifically, a non-lethal harvest of palm mesh fibers was carried out in a farm located in Ismailia, Egypt; this species is classified as having "least concern" status in the IUCN Red List Assessment.

#### Phase one: fibers treatments

**Figure 6:**
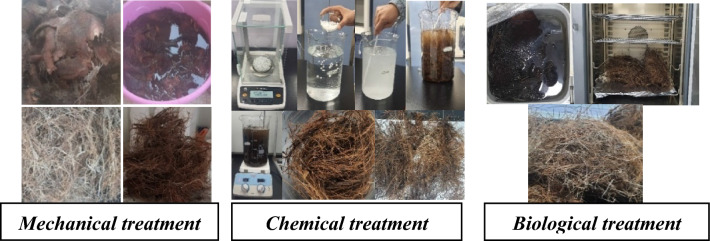
Mechanical, chemical, & biological fibers treatments (developed by author, 2023).

The male date palm mesh was collected during the harvesting season of the date palm trees form a farm in Ismailia, Egypt all the fibers were gathered from Sukkari date palms as shown in Fig. 6 below, then 3 types of treatments was used to treat the fibers:

Mechanical treatment: the date palm mesh was soaked in water for 24 h to ensure saturation, then hand-separated and dried at room temperature for another 24 h before being cut into 60 mm pieces. The chemical treatment: the date palm mesh was soaked for 1 h in 1% NaOh at 100 °C, then it was chopped to 60 mm pieces which is the most common size used in literature. While the Biological treatment: the retting process was used to extract the fibers through which the mesh was soaked in water for 20 days to rot, after that it was cleaned, and the fiber was separated and dried under sunlight. Then the fiber was kept at 100 °C for 24 h for partial removal of moisture.

#### Phase two: concrete mixture

Ten M30 mixtures of plain concrete were created with the addition of male date palm fibre with a 0%, 0.4% (30 gm), 0.6% (45g m), & 0.8% (60 gm) respectively for each type of treatment to create a total of 150 samples. There sample sizes varied to suit the different type of tests, there were 30 cylindrical samples that measured 10 × 10 × 20 cm and was used for the split cylinder tests, 30 cuboid samples that measured 10 × 10 × 50 cm and were used for the flexural strength tests, while 90 cube samples that measured 10 × 10 × 10 cm and was used for the rest of the tests. The material properties of the components used in the concrete mixtures are listed in Table 1 below.

**Table 1:** The concrete mixture components properties according to the supplier (developed by author, 2022).

Cement	Physical & mechanical properties	Setting time (min)	≥ 60
		Expansion (mm)	≤ 10
		Compressive strength (MPa)	≥ 42.5
	Chemical properties	Loss if ignition	≤ 5%
		Insoluble residues	≤ 5%
		Sulphate (SC3)	≤ 3.5%
		Chloride content (CL-)	≤ 0.1%
Sand	Physical properties	Specific gravity	2.5
		Fineness modulus	2.46
		Type of sand	Desert sand
		Sand size	Medium sand
Gravel	Physical properties	Specific gravity	2.5
		Gravel size (mm)	4.75 ≥ x ≥ 9.5
		Color	Grey
		Shape	Angular
Water	Good potable water available at BUE university’s laboratory free of debris		

**Figure 7:**
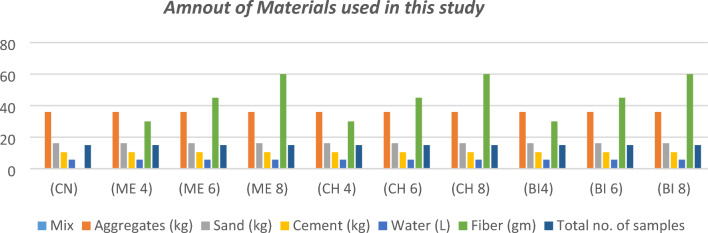
Materials used in the concrete mixtures (developed by author, 2022).

The concrete mixtures were designed according to the Egyptian standard guidelines by using the materials listed in Fig. 7 below.

### Experimental tests

**Figure 8:**
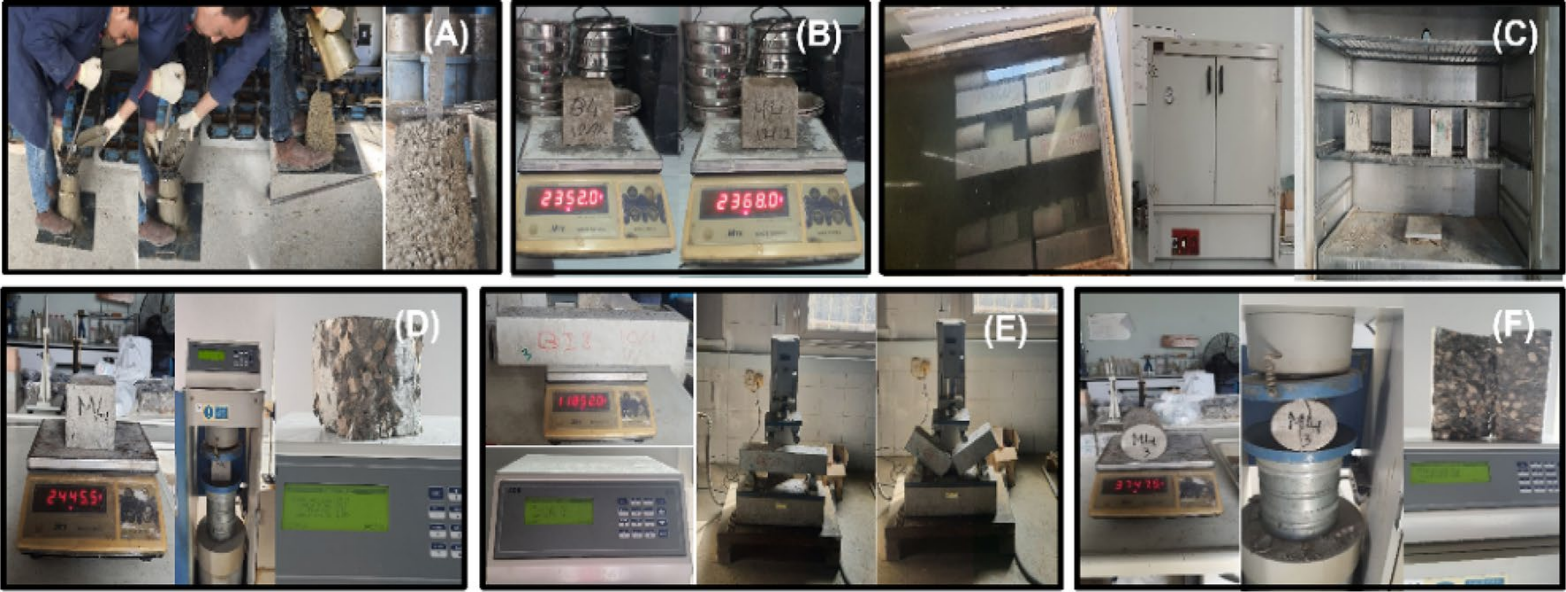
(**A**) Slump test, (**B**) unit weight, (**C**) absorption rate, (**D**) compressive strength test, (**E**) flexural strength test, and (**F**) split cylinder test (developed by author, 2023).

*The slump test:* The concrete slump test indicates the consistency of a concrete mixture. This test is done on the fresh concrete after the mixing and is presented in Fig. 8 below.

*The unit weight:* The weight of concrete required to fill a container of a defined volume is referred to as the unit weight, the unit weight was measured for a 10 × 10 × 10cm cube.

*The absorption rate:* was measured at the age of 7 and 28 days, through measuring the sample weight before curing then after 7 days, and after 28 days.

*The Compressive strength:* Concrete’s ability to withstand loads before failing. It was measured for the samples at the age of 7 and 28 days using compression testing machine.

*Flexural strength test:* Flexural strength is a form of measuring concrete tensile strength since it measures the ability of a plain concrete beams and slabs to withstand bending failure. It was measured for the samples at the age of 28 days using bend test machine.

*Split cylinder test:* This test is used to determine the lateral tensile stress of concrete; it was measured for the samples at the age of 28 days using compression testing machine.

#### Thermal conductivity test

**Figure 9:**
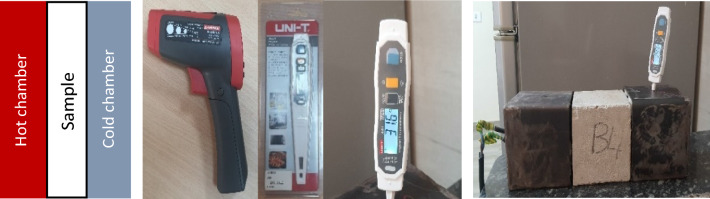
The box method & the Thermometers used to measure the temperature in the box method (developed by author, 2023).

The box method was used to calculate the heat transition as shown in Fig. 9. The device contains one hot chamber that had a constant temperature output level which is approximately 732.7 C° (measured by Am probe), the heat transition from the hot chamber to the cold chamber was calculated through measuring the temperature of the cold chamber before adding the heat source then measuring it again after 25 min to measure the amount of heat transferred through the samples.

## Results and findings

The findings will be presented into two different levels; the experiment conducted, and the observational results noted during the experiment. Slump, absorption, unit weight, compressive strength, flexural strength, tensile strength, and thermal conductivity are the experimental tests of the concrete’s samples with different treatments and percentages of fibres, while comparing them to the control samples. The maximum, average, and minimum experimental results of the tests are illustrated in Table 2 below.

**Table 2:** The maximum, average, and minimum experimental results of the conducted tests (developed by author, 2023).

Curing	Value	Slump | mm		Unit weight |1 sample | Kg		Absorption |2 samples | %		Compressive strength | 6 samples | MPa		Modulus of rupture | 3 samples | Pa		Lateral strength| 3 samples | 2Pavg /πøL (Pa)		Thermal conductivity| 1 sample | C°	
7 DAYS	Max	M6	10	B4	2.4	M8	3.8	M8	27.4						
	Ave	M4/B4	50	C4	2.4	B4/B6	5.9	M6	23.9						
	Min	C8	105	M6	2.4	C4/C8	8.1	C8	8.3						
28 DAYS	Max					B8	5	B8	37.6	B4	9.95	C6	3.97	C4	3.5
	Ave					M4/M6	5.8	M4	31.6	M6	6.6	M6	3.06	B6	5.2
	Min					B4	7.6	C4	14.6	M8	4.35	C4	1.56	M8/B8	6.3

Table 3 below illustrates the comparative analysis between the different percentages and treatments of fibres additions to the concrete mixtures, noting that the durability, cost, environmental impact, and human health impact were evaluated by the authors.

**Table 3:** Comparative analysis results (developed by author, 2023).

	CN	Mechanically treated fibres	Chemically treated fibres	Biologically treated fibres						
		M4	M6	M8	C4	C6	C8	B4	B6	B8
Absorption	H	L	L	L	L	L	L	L	L	L
Compressive strength	L	H	H	H	L	H	L	H	H	H
Thermal conductivity	H	L	L	L	L	L	L	H	L	H
Workability	H	H	M	L	H	M	L	H	M	L
Colour	Light grey									
Surface	VS	VS	S	SR	VS	S	SR	VS	S	SR
Cracks	No cracks									
Durability	Durable									
Cost	M	C	C	C	E	E	E	M	M	M
Type of construction	Permanent & temporary									
Environmental impact	H	L	L	L	H	H	H	L	L	L
Availability of raw material	Available									
Human health impact	H	L	L	L	H	H	H	L	L	L

*H* high, *L* low, *M* moderate, *C* cheap, *E* expensive, *VS* very smooth, *S* smooth, *SR* slightly rough.

According to this experimental study, The M6 samples has successfully reduced the human health impact and environmental impact while providing lower thermal conductivity and better strength.

## Conclusion

This study investigated the possibility of using different treatment methods to treat male date palm fibers in order to utilize them in the plain concrete mixtures in order to improve it physical and mechanical properties and produce ecofriendly concrete through minimizing the harmful effects of improper disposal of organic waste such as the date palm residues, while decreasing the use of cement content since adding the fibers helps in combining the plain concrete’s components and slightly increase the volume of the total mixture. The research experiment added three different percentages of date palm fibers to identify the best combination of treatment and percentages to achieve the ideal prototype with the best physio-mechanical properties. Unlike Alatshan et al.^15^ work that stated that with increasing fiber percentage and length, the compressive strength decreased while the flexural strength increased. The research finding proved that the best properties were of the samples that included 0.6% mechanical treated fibers, while highlighting that the chemical treatment produced the worst physio-mechanical properties of the concrete samples.

## Data Availability

Finally, to complete their investigation, the authors just collected the bare minimum of specimens. All data are available from the corresponding author on reasonable request.
